# 2D Kinematic Analysis of the *Esbarrada* and *Volta Sobre Patas* Manoeuvres of Criollo Breed Horses Competing in *Freio de Ouro*

**DOI:** 10.3390/ani14162410

**Published:** 2024-08-20

**Authors:** Gino Luigi Bonilla Lemos Pizzi, Karina Holz, Éverton Augusto Kowalski, Priscila Fonseca Ribeiro, Roberta Blake, Charles Ferreira Martins

**Affiliations:** 1 Faculdade de Zootecnia, Universidade Federal de Pelotas, Capão do Leão 96160-000, Brazil; gino_lemos@hotmail.com (G.L.B.L.P.); evertonequinocultura@gmail.com (É.A.K.); priscilafri@hotmail.com (P.F.R.); 2 Faculdade de Veterinária, Universidade Federal de Pelotas, Capão do Leão 96160-000, Brazil; karinaholz06@gmail.com; 3 Writtle School of Agriculture, Animal and Environmental Sciences, Anglia Ruskin University, Writtle, Chelmsford CM1 3RR, UK; 4 Departmento de Clínicas Veterinárias, Universidade Federal de Pelotas, Capão do Leão 96160-000, Brazil; martinscf68@yahoo.com.br

**Keywords:** biomechanics, kinesiology, performance assessment

## Abstract

**Simple Summary:**

Equestrian sports have evolved beyond traditional disciplines, embracing specialised events that showcase breed-specific abilities. The Criollo horse breed, a product of centuries of genetic refinement, stands out in South America and internationally, particularly in events like the *Freio de Ouro*. This competition highlights the breed’s versatility and skills, emphasising manoeuvres like the *esbarrada* and *volta sobre patas*, which demonstrate agility, coordination, and balance. Despite the breed’s success, there is a lack of quantitative kinematic studies on these manoeuvres and their relation to horse morphology. Our study aims to fill this gap by using kinematics to analyse the *esbarrada* and *volta sobre patas*, exploring any connections between static goniometry and manoeuvre dynamics. Understanding these movements not only enhances our appreciation of equine biomechanics but also informs breeding and training practices, ultimately benefiting both horses and riders in various equestrian disciplines.

**Abstract:**

The *esbarrada* and *volta sobre patas* manoeuvres are critical in Criollo horse equestrian activities, yet their kinematics remain largely unexplored. This observational study aimed to kinematically describe the manoeuvres in Criollo breed horses and explore the relationship between static goniometry and dynamics. A 2D kinematic analysis was conducted on 31 Criollo horses performing the manoeuvres using high-speed cameras. Joint angles were measured using static goniometry and analysed in relation to dynamic performance. The *esbarrada* covered a distance of 4.28 ± 0.99 m in 1.15 ± 0.11 s at a velocity of 3.77 ± 0.55 m/s. Joint angles showed a mean fetlock extension of 75.4° ± 5.3° and hock flexion of 43.2° ± 4.1°. In the *volta sobre patas*, significant differences were found in turn duration (first: 0.96 ± 0.07 s, second: 1.12 ± 0.09 s, *p* = 0.03) and thoracic limb suspension (first: 0.23 ± 0.02 s, second: 0.28 ± 0.03 s, *p* = 0.02). Static goniometry indicated that limb conformation did not significantly correlate with protraction angles during the *esbarrada* (*p* = 0.27). The *volta sobre patas* demonstrated prolonged duration and increased thoracic limb suspension in the second turn. Also, the findings suggest that static conformation does not predict dynamic limb movement during the *esbarrada*.

## 1. Introduction

Equestrian sports, traditionally centred on classic disciplines such as show jumping, dressage, and cross-country, have been expanding their scope to more specialised disciplines with high cultural appeal at an international level, focusing on breeds with specific abilities around the world since the Industrial Revolution in the mid-19th century [1]. Adelman and Thompson [2] described that equestrian culture accompanies important social transformations introduced by post-modernity, equally affecting global and local scenarios in the cultural and economic spheres related to the species.

In South America, the Criollo breed is notable for its genetic selection and modification, establishing a distinct racial standard. Prominent in various competitive events, the *Freio de Ouro* is particularly significant for its role in selecting Criollo horses with exceptional zootechnical characteristics [3]. This competition, emphasising practical cattle work skills, requires agility, coordination, and balance. According to the Associação Brasileira de Criadores de Cavalos Crioulos (ABCCC) regulations (2015), Criollo horses, originating from Spanish Andalusians, are balanced and harmonious, with females weighing 400–450 kg and standing at 1.38–1.48 m and males at 1.40–1.50 m. They have a structure suited for rapid stops and agile direction changes, essential in cattle handling and reflecting cultural traditions [4,5,6]. To qualify for the finals, horses and riders must undergo rigorous testing across southern Latin America, combining morphological and functional assessments such as reining, cutting, and cattle handling, which test their abilities and athleticism [7,8,9]. The *Freio de Ouro* is intrinsically characterised by its multifactorial nature, which requires the harmonious combination of several physical and behavioural elements of individuals of the Criollo breed, such as agility, obedience, balance, and muscular resistance of the horse–rider system [10]. This comprehensive competition highlights the versatility of the equestrian discipline while also evaluating the team’s ability to face challenges ranging from executing precise manoeuvres to demonstrating physical and mental endurance [11]. Typically, the sequence includes the demonstration of specific gaits, such as the walk, trot, and canter, followed by a smooth transition to manoeuvring and cutting. Specific manoeuvres are key features, such as the *esbarrada*, which entails a rapid halt from a gallop, with the horse sliding on its hind legs while maintaining stability and precision, and the *volta sobre patas*, which involves intricate turns and latero-flexions of the spine, pivoting around a hind leg, lifting its forequarters to execute a smooth, controlled spin. These manoeuvres test and showcase the horse’s dexterity, coordination, and balance, which are crucial for effective cattle handling, and the evaluations focus on the animal’s responsiveness and posture and the rider’s control.

While similar manoeuvres can be found in other equestrian disciplines (e.g., the sliding stop and spin in reining), the Criollo-specific competitions highlight the breed’s unique capabilities. Competitors in the *Freio de Ouro* and similar disciplines like Western reining share similarities in performing precise, rapid manoeuvres and showcasing agility and responsiveness to the rider’s cues. However, Criollo horses also demonstrate a distinct blend of speed, endurance, and cattle-herding capabilities, which differ from the elegance and extended gaits emphasised in dressage horses. This highlights their unique adaptation to the diverse demands of ranch work and competitive scenarios. Despite the presence of these manoeuvres, there is a lack of descriptive quantitative kinematic studies and analyses of the influence of morphology on such movements within the existing literature on equine biomechanics. Pimentel et al. [12] observed that linear and angular morphometric measurements of Criollo breed horses explained 83% of the variation in the morphological score in the context of the *Freio de Ouro*, demonstrating a significant association between these characteristics and the judges’ subjective assessment in a morphological analysis. However, there are no studies that associate this angular measurement with the dynamics of the manoeuvres.

This study aims to fill this gap by presenting a detailed kinematic analysis of the *esbarrada* and *volta sobre patas* movements and determining whether there is an association between static goniometry and the dynamics of these manoeuvres. By focusing on these specific movements, this study will provide new insights into the functional morphology of the Criollo breed, supporting breeders and trainers in optimising performance and maintaining the breed’s distinctive characteristics. We hypothesise that a detailed kinematic analysis of the *esbarrada* and *volta sobre patas* movements in Criollo horses will reveal a significant correlation between static goniometry measurements and the dynamics of these manoeuvres, enabling the optimisation of training and selection techniques to enhance performance and maintain the distinctive characteristics of the breed in both traditional and competitive settings. The findings will not only advance scientific knowledge but also have practical implications for improving training techniques and enhancing the overall quality of competitive Criollo horses. This study is of significant value to the industry, offering potential improvements in the selection and training processes that could enhance the breed’s performance in traditional and competitive settings.

## 2. Materials and Methods

### 2.1. Study Design

All procedures performed were approved by the Ethics Committee on the Use of Animals (CEUA) of the Federal University of Pelotas (UFPel) under registration number of 51839-2019. 

### 2.2. Experimental Design

From an initial pool of 35 animals, 31 Criollo breed horses (*Equus caballus*) were evaluated, aged between 5 and 10 years (7.5 ± 2.6 years), 22 males and 9 females, with an average weight of 428.41 ± 24.47 kg and wither height of 1.42 ± 0.01 m (Table S1). These individuals belonged to training centres in the southern microregion of the state of Rio Grande do Sul, Brazil. All selected horses were trained and competitors in the *Freio de Ouro* discipline. The training regimen of these individuals comprised a minimum period of two years of weekly exercise routines, with their qualifications for competitions starting at 24 months of age. Each training centre has its rider responsible for training and performs similar routines at least five times a week. This selection ensures that the horses analysed are representative of elite competitors in the breed’s most prestigious event. The inclusion criteria were stringent, focusing on horses that demonstrated training and performance levels consistent with high-stakes competition, including several finalists. Four horses were excluded from the study because they did not perform the manoeuvres in a straight line within the study field during data collection, nor did they meet the competitive standards required for accurate kinematic analysis. The sample size was determined based on these stringent criteria to ensure the reliability and accuracy of the analysis. This criterion not only highlights the breed’s capabilities but also ensures that the selected animals are the best exemplars of the movements under study.

Collections were carried out in equestrian training centres in June 2022. All animals were subjected to the same previously defined experimental and environmental conditions. Furthermore, all equestrian surfaces comprised a sand-based soft floor, mimicking the competition arenas in the discipline. Before the kinematic analysis, the horses were subjected to a specific clinical examination of the locomotor system to determine the health status of the population sample. The evaluation was carried out by an experienced clinician, who found a grade 0 on the AAEP scale (American Association of Equine Practitioners) regarding the presence of lameness in the animals included in the study. All individuals were considered sound, without lameness or other musculoskeletal illnesses, and therefore able to be included in the investigation.

### 2.3. Kinematic Data Collection

The positioning of markers and the setup of the study field were based on the methodology outlined by Torres-Pérez et al. [13]. Thirty retroreflective markers (30 mm in diameter) were positioned and fixed with double-sided tape by the same operator on the right and left sides of the animals in the anatomical region referring to the bony protuberances of the middle third of the facial crest, lateral apex of the atlas wing (C1), and dorsal to the spinous process of the first sacral vertebra (S1) and on the anatomical eminences of each limb: thoracic limbs—tuberosity of the spine (scapula), cranial aspect of the greater tubercle (humerus), lateral tuberosity at the insertion of the lateral collateral ligament of the joint of the elbow (radius), styloid process (ulna), lateral collateral ligament of the fetlock (III metacarpal bone), and coronary line on the podophalangeal axis (middle phalanx); hindlimbs—coxal tuberosity, greater trochanter (femur), lateral condyle (tibia), lateral malleolus (fibula), lateral collateral ligament of the fetlock (III metatarsal), and coronary line on the podophalangeal axis (middle phalanx).

The study field was 10 m long and 3 m wide, demarcated by cones for easy identification. By the sides, there was an area to allow the animals to slow down and reposition themselves. A high-speed camera (iPhone 12 Pro Max, Apple Corporation, Cupertino, CA, USA) collecting at 240 fps and 1280 × 550 resolution was used, levelled horizontally by a fixed tripod 1 m high and positioned between 7 and 10 m from the centre of the platform. A 72 W LED light was also positioned above the camera to activate the reflectivity of markers placed on subjects. Exactly in the centre of the field, a 1 m ruler was placed in horizontal and vertical positions to calibrate the software system. This configuration was standard for all training centres, being reproduced equally in each location.

Before data collection, a walk and trot warm-up was performed for a period of 10 min. Then, the riders led the animals in a straight line exactly to the centre of the study field, starting from a point located 20 m away. 

For each horse, two slow-motion videos, with an average duration of 10 s, of each manoeuvre from both the left and right sides were analysed, totalling 124 videos. The same examiner, an expert with extensive experience in equestrian biomechanics, reviewed all videos to ensure consistency and objectivity. This examiner verified that the movements closely mimicked competition-level execution. Specifically, for the *esbarrada*, the manoeuvres had to be executed in a sagittal plane with a straight-line trajectory, avoiding lateral deviations and axial rotations. For the *volta sobre patas*, the horses began perpendicularly to the camera and executed turns around their axis, returning to their initial position upon completion. This rigorous selection process ensured that the movements analysed are ideal representations of the breed’s capabilities in competitive scenarios. The use of competition guidelines to evaluate the manoeuvres guarantees that the kinematic data collected are reflective of the highest standards in Criollo horse performance.

After collection, the videos were processed and analysed using the Quintic Biomechanics^®^ v33 2D motion analysis system (Quintic Consultancy Ltd., Coleshill, Birmingham, UK), where the variables obtained were tested and quantified.

### 2.4. Kinematic Variables

#### 2.4.1. Esbarrada

The measurements of the *esbarrada* were assessed with a focus on the initial frames capturing the ventral pelvic engagement towards the abdomen. Subsequently, the initial contact of the pelvic limbs with the ground until the moment of cessation was analysed, thus delineating a complete and effective *esbarrada* in the sagittal plane and in a straight line. The variables analysed during the movement comprised temporal, linear, and angular values, being measured individually for the fore- and hindlimbs on both sides as well as in the vertebral segments. Initially, measurements were taken of the length (m) and duration of the *esbarrada*(s) based on the first frame of contact of the pelvic limb(s) on the ground until the sliding completely stopped. The length/time ratio was performed to determine the speed of the manoeuvre.

**Figure 1:**
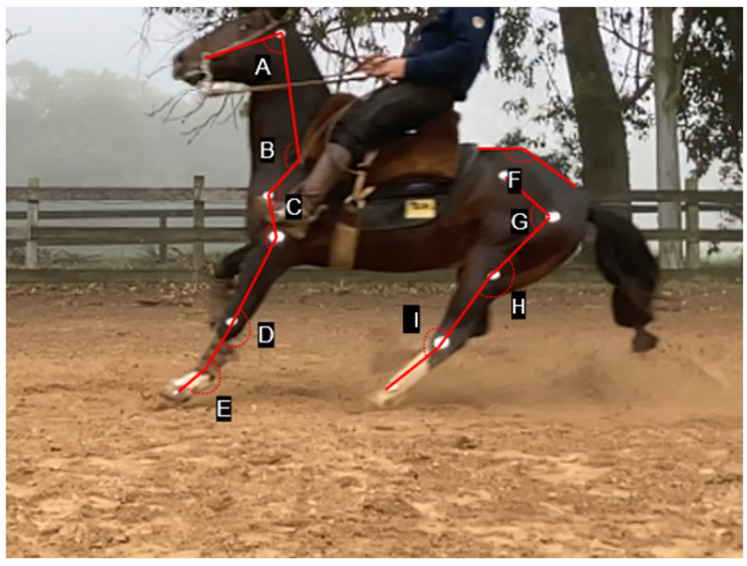
The moment of engagement of the pelvic limbs before hoof–ground contact during the execution of the esbarrada manoeuvre on Criollo breed horses competing in the Freio de Ouro, with the representation of the kinematic angles analysed: head angle (A), shoulder (B), elbow (C), carpal (D), forelimb fetlock (E), lumbosacral (F), hip (G), stifle (H), and hock (I).

Moreover, the angular variables investigated consisted of the protraction angle (°) of the thoracic and pelvic limbs at the time of engagement (maximum protraction of the hindlimb during the manoeuvre) of these segments for the manoeuvre (Figure 1). In addition to this, at the same moment, the angle of the head was also measured between the central point of the atlas wing, with vertices between the facial crest and the scapular marker as well as each joint of the thoracic limbs (shoulder, elbow, carpal, and forelimb fetlock) and pelvic area (lumbosacral, hip, stifle, and hock). The angular measurement referring to the metatarsophalangeal joint was not included in the study due to the impossibility caused by artefacts in the images arising from the presence of sand during the animals’ execution of the manoeuvre.

Empirically, it is believed that animals exhibiting greater angulation during static inspection demonstrate increased protraction angles, consequently enhancing limb engagement during movements like the *esbarrada*. To descriptively test this effect, we compared the protraction angles obtained from the thoracic and pelvic limbs and the kinematic angular values of each joint within these segments. This comparative analysis aimed to elucidate the relationship between static angulation and dynamic limb movement, providing insights into the biomechanical mechanisms underlying improved engagement during locomotion. In order to elucidate the impact of joint goniometry on the execution of the *esbarrada*, angular measurements were obtained in a stationary position for the specified joints and subsequently compared with the angular values recorded during the performance of the manoeuvre, where the averages found were as follows: shoulder 112.35 ± 11.37°; elbow 139.65 ± 13.14°; carpal 183.26 ± 4.72°; forelimb fetlock 214.33 ± 13.68°; head 107.47 ± 9.88°; lumbosacral 128.40 ± 13.06°; hip 99.40 ± 8.95°; stifle 154.68 ± 11.96; and hock 154.73 ± 7.86°. 

#### 2.4.2. Volta Sobre Patas

**Figure 2:**
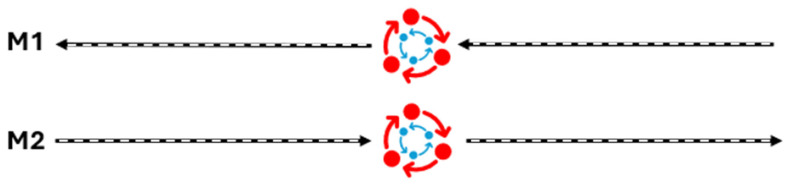
The scheme representing the two moments (M1 and M2) where the volta sobre patas was analysed, with Set 1 (red) being the first pair of spins the same side and Set 2 (blue) being the pair of contralateral spins (the sizes of the circles and arrows are only illustrative and do not reflect the circumference made by the animals).

For the *volta sobre patas* manoeuvre, angular and temporal variables were measured due to the impossibility of obtaining linear measurements given the three-dimensional characteristic of the movement. In the present study, the *volta sobre patas* manoeuvre was structured to mirror the specific requirements expected from Criollo breed horses in competitive performances. Set 1 entailed a sequence of two full turns executed consecutively to one side, followed by Set 2, which comprised two turns in the opposite direction, each set performed at distinct moments (M1 and M2), resulting in a total of four repetitions encompassing eight turns (Figure 2). The duration (in seconds) for each set of *voltas* was meticulously recorded at both moments. Concurrently, the average suspension time of both forelimbs operating simultaneously was assessed during each set of turns. 

**Figure 3:**
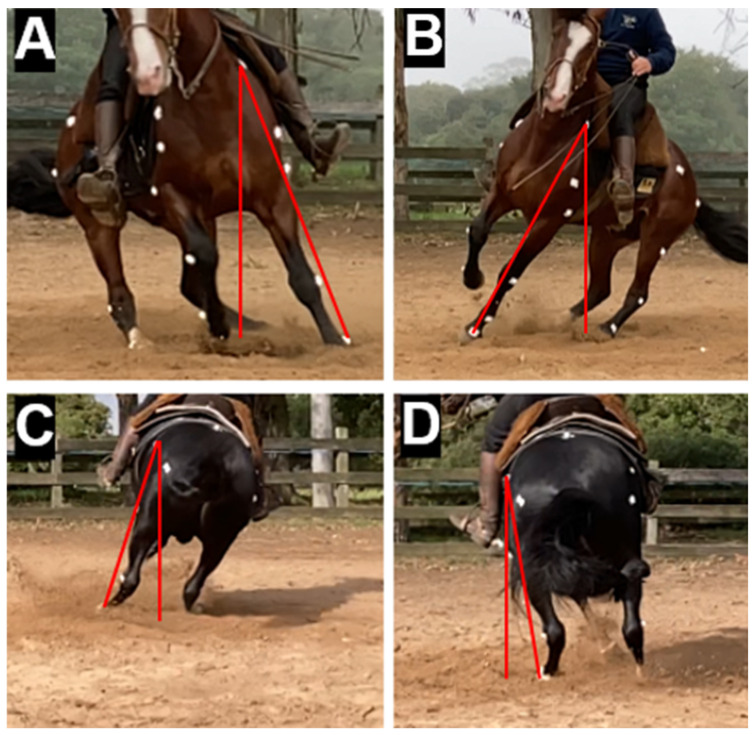
The angular measurement of forelimb abduction and adduction movements ((**A**) and (**B**), respectively) and the hindlimbs ((**C**) and (**D**), respectively) in Criollo breed horses competing in the Freio de Ouro during the volta sobre patas manoeuvre.

Also, the support and suspension times of each fore- and hindlimb were obtained during an entire turn. Finally, the adduction and abduction angles of the thoracic and pelvic limbs were measured when perpendicular to the camera. To determine the quantitative parameters of these variables, both sets were observed at the same moment (M1), and the average values of the fore- and hindlimbs on the outer side of the circle (OFL and OHL, respectively) and of the contralateral limbs on the internal side of the turn were taken, where they were called the inner forelimb (IFL) and pivot hindlimb (PHL)—the latter due to its characteristic of serving as a support axis during the manoeuvre. In the forelimbs, the angles were measured at the vertex between a vertical line and another straight line from the scapular marker to the one at the fetlock level. In the hindlimbs, a similar measurement was performed, and the straight line in the segment was between the marker referring to the thigh tuberosity and the pelvic fetlock (Figure 3). All analyses included frames chosen where the animal was closest to the centre of the study field to avoid perspective and parallax errors. 

Due to the impossibility of obtaining kinematic angular data from the thoracic and pelvic limb joints during the *volta sobre patas* movement as well as the absence of protraction in this type of motion, the comparative analysis with static measurement was not performed.

The selected parameters for measuring the *esbarrada* and *volta sobre patas* movements were carefully chosen due to their crucial significance in evaluating the biomechanics and performance quality of Criollo breed horses during competitive manoeuvres. The measurements of length, duration, speed, and angular angles, such as protraction angle and joint goniometry, were specifically chosen to capture the precise mechanics and joint dynamics involved in those manoeuvres. These parameters were deemed significant, as they offer a comprehensive understanding of the joint angles, limb support and suspension times, and overall movement quality, which is crucial for assessing the precision, balance, and agility required in these specialised equestrian manoeuvres.

### 2.5. Statistical Analysis

For each kinematic variable, the average values and standard deviation between the videos collected from each side during the manoeuvres were considered, and subsequently, the average value between the right and left sides was adopted for descriptive statistics. Aiming to compare the static and dynamic angular variables during the *esbarrada*, these were subjected to multiple linear regression analysis with the static goniometric data and the fore- and hindlimb protraction angles. In the temporal variables of the *volta sobre patas*, the total times of the first and second sets of turns were compared to describe the change over time in both moments as well as the time of simultaneous suspension of the thoracic limbs. Initially, the data were tested for normality using the Shapiro–Wilk test. For parametric data, means were compared using the paired T-test, while non-parametric data were analysed using the Wilcoxon test. For all hypotheses, a value of *p* ≤ 0.05 was assumed. Statistical analyses were performed using SPSS^®^ IBM v20 software. Descriptive values of the final means and standard deviation (SD) of all variables, except for the total and simultaneous thoracic suspension times of the *volta sobre patas* sets, were obtained for each thoracic and pelvic limb.

## 3. Results

The descriptive statistics of the kinematic variables of the esbarrada and volta sobre patas of the 31 animals included in the study are shown below.

### 3.1. Esbarrada

The sliding length was 4.28 ± 0.99 m long, with a duration of 1.2 ± 0.1 s and a speed of 3.77 ± 0.55 m/s. In the angular kinematic variables, the head angle was 81.5 ± 6.9°, and protraction angles were 27.1 ± 2.8° and 33.0 ± 4.2° for the thoracic and pelvic limbs, respectively (Table S2). No significant relation was determined between static goniometry and the protraction angles of the fore- and hindlimbs during the moment of limb engagement (maximum protraction during the manoeuvre) (*p* > 0.05). The descriptive angular values of the joints for each thoracic and pelvic limb, as well as the total average value, are described in Table 1 and Table 2 and Tables S3 and S4.

### 3.2. Volta Sobre Patas

In this section, we present the average data of the temporal and angular kinematic variables of the vota sobre patas manoeuvre (Table 3 and Table S5). The times of the sets of turns differed in both moments (M1, *p* = 0.023; M2, *p* < 0.001), as did the time of simultaneous suspension of the thoracic limbs (*p* = 0.013 and 0.023, respectively). The kinematic values of the support and suspension times of the thoracic and pelvic limbs can be seen in Table 4 and Table S6. The limbs maximum adduction and abduction angles of the outer and inner thoracic and pelvic limbs are shown in Table 5 and Table S7.

## 4. Discussion

### 4.1. Esbarrada

The *esbarrada* is a complex manoeuvre and consists of an abrupt stop of the horse at high speed, symbolising the instantaneous response to the rider’s direction [14]. The animal approaches through a gallop in a physical space that comprises around 20 m. The results demonstrate that the animal slides a distance of 4.28 ± 0.99 m with a time of 1.2 ± 0.1 s at the first moment of contact with the surface. The speed decreases due to the deceleration that the movement promotes and may vary depending on the intrinsic factors of different equestrian surfaces, such as shear force, depth, and composition material [15,16].

The starting point of the manoeuvre begins with the ventroflexion of the lumbosacral joint, reaching average angular values of 123.7 ± 5.7°, allowing for the engagement of the pelvic limbs ventrally to the trunk, which protracts 33.0 ± 4.2° due to the flexor action on the joints of this segment. The kinematic data in the present study quantitatively characterise the patterning of the *esbarrada* manoeuvre. In the animals evaluated, this movement was performed with different dynamic biometric aspects, with particularly dissimilar characteristics when compared to genetic groups in similar sports (e.g., Quarter Horse), such as an erect neck and flexion in the atlantooccipital region, reaching average indices of 81.5 ± 6.9°, allowing for the dorsiflexion of the spine to engage the pelvic limbs. It is notorious that the protraction of the thoracic limbs is facilitated by the extensor action in the joints of these segments, with a bilateral average of the shoulder of 117.7 ± 6.4°, a value of 130.2 ± 6.5° for the elbow, and values of 180.9 ± 3.4° for the carpal and 220.3 ± 6.4° in forelimb fetlock joints. These quantitative objective values indicate extensor activity in the limb, allowing for the protraction of 27.1 ± 2.8° for the stabilisation of the thoracic limbs during the slide. Numerically, the pelvic limbs showed a protraction of 33.0 ± 4.2° compared to the angular values observed in the thoracic limbs when executing the *esbarrada* manoeuvre. These dynamic values characterise that the degree of pelvic protraction is directly related to flexion actions mainly of the more proximal joints, facilitating the engagement of the limb in the region ventral to the abdomen.

Despite the variability in the morphometry of the animals used in this study recorded at the station, no significant relations (*p* > 0.05) were observed with the kinematic values of the protraction of the fore- and hindlimbs, indicating that extrinsic factors such as training, the physical conditioning and experience of the trainer/rider, and the characteristics of the equestrian surfaces determine the skill in the movement of this manoeuvre.

The sliding stop is a manoeuvre with similar features to the *esbarrada*, characteristic in the reining, a Western riding discipline. In this equestrian sport, the horse is subjected to abrupt deceleration from a fast gallop to a sliding stop, maintaining balance and sliding on the pelvic limbs [17], a manoeuvre like the one performed by horses in the Criollo breed despite having a striking visual characteristic of the head being kept in a lower position, with the neck being positioned cranioventrally extended. Within this context, the descriptive data presented fill an existing scientific gap, serving as a basis for different equestrian disciplines where the execution of this movement is required. Although numerical variations occurred in the observations captured during the kinematics, these did not appear when comparisons were made on both the left and right sides, demonstrating coordination during the biomechanics of the *esbarrada*.

It is important to consider the fact that the *esbarrada* requires repositioning the spine in an extended position, with great participation from the shoulder and fetlock region, keeping the thoracic limbs in a considerable degree of protraction (27.06 ± 2.81°) and allowing for a greater axial diagonalisation, thus facilitating pelvic engagement for sudden braking. Furthermore, the erect position of the neck and the reduced flexion of the head (81.5 ± 6.9°) allow for greater stability and accuracy for the animal to bump and then return to the station after stopping. The position of a horse’s head and neck significantly affects the characteristics of its back and stride [18,19]. When the neck is extended, there is an increase in extension in the thoracic region and flexion in the pelvic and lumbar region, while a lowered neck produces the opposite effect [18]. This is due to the influence of the neck muscles on head movement and posture maintenance [20]. In dressage, different head and neck positions can lead to changes in the horse’s movements, with an extremely high neck position potentially increasing the risk of injury [21]. This information highlights the importance of considering the position of the horse’s head and neck in training and rehabilitation programs as well as being in line with the kinematic aspect that Criollo breed horses perform during the moment the limbs are engaged in the *esbarrada* manoeuvre, thus maximising its biomechanical efficiency.

The range of movement during ventroflexion in movements such as the *esbarrada* occurs through the lumbosacral joint (123.7 ± 5.7°), which is the point of greatest intervertebral mobility among the thoracic, lumbar, and sacral segments due to characteristics such as increased thickness and decreased height of the intervertebral disc, a wide divergence of the dorsal spinous processes, poorly developed interspinous ligament, the absence of supraspinous ligaments, and the vertical orientation of the articular facets [22,23,24,25]. The biomechanics of this axial joint segment allows for a greater range of motion in the hip, especially at the hip joint, promoting a consequent accentuated mobility of the distal joints in the pelvic limb [26]. Hodson et al. [27] had already described the relationship between the range of hip movement and greater protraction angles of the pelvic limb in gaits such as a walk, a fact that can be corroborated for manoeuvres like the *esbarrada*, as observed in the present study. These kinematics allow for the tarsal region to promote the main ventral engagement with the abdomen, directly influencing pelvic protraction.

### 4.2. Volta Sobre Patas

This is a manoeuvre performed where the horse spins around its axis through lateroflexions of the spine to both sides, completing a pair of circles [6]. In the present study, although the total manoeuvre time was numerically similar, differences were observed at both moments (M1: *p* = 0.023; M2: *p* < 0.001), when Sets 1 and 2 were considered. A plausible hypothesis lies in the complex interaction between biomechanical and neuromuscular factors during the execution of these movements. During the first set of turns, it is possible that the horses are initially adapting to the specific kinetic and kinematic demands associated with the manoeuvre. A plausible explanation for this divergence lies in the hypothesis of motor coordination since athletic performance in this manoeuvre is the result of coordinated muscular actions, influenced by the composition of muscle fibres and the precise integration between the nervous system and muscular contraction [28]. 

Initially, it is crucial to consider that performing repeated *voltas sobre patas* requires precise coordination between different muscle groups and neuromotor systems. During the execution of the first set, the muscles responsible for the stabilisation and lateral propulsion of the axial segments are relatively less activated, resulting in an initially more optimised biomechanical efficiency. However, as the sequence of turns progresses to the second set, there is a need to quickly change direction, activating muscle groups responsible for the contralateral movement, leading to a slight decrease in the effectiveness of the stabilisation and propulsion mechanisms that can be observed as increasing the time in Set 2. Within the results observed, the thoracic suspension time can be translated as a synchronisation of the movements of the thoracic limbs and greater stability for a new sequence of lateral propulsions of these segments, allowing for the suspension time in the second set to be prolonged, thus elucidating the increase in this duration compared to the first set in both M1 and M2 (*p* = 0.013 and 0.023, respectively). It is necessary to remember that this manoeuvre is performed after other athletic demands throughout the *Freio de Ouro* event. This rapid neuromuscular activity can be influenced by the accumulation of metabolites, such as lactate, and the decreased availability of anaerobic energy, resulting in decreased muscle responsiveness and coordination [29]. This effect has already been observed in humans, where muscle fatigue led to a change in the organisation of movement, and other researchers suggest a similar effect in horses, highlighting the temporal sequence of fatigue and changes in coordination [30,31]. Consequently, executing the turns in the second set, regardless of the moment (M1 or M2), may require prolonged and more intense muscular activity to maintain the necessary stability and boost, thus prolonging the total duration of the movement. Johnston et al. [32] concluded that both load and fatigue can alter locomotor patterns in horses, leading to an increase in kinematic variables, such as joint excursion and stride length, corroborating the changes observed in temporal variables observed in the present study. Therefore, it is important to consider that previous experience and motor learning also play a significant role in this temporal disparity. Horses from different equestrian disciplines can learn and adapt their movement strategies based on previous experiences through the unique structure and coordination of the limbs, refining their biomechanical efficiency over time [33]. In short, the difference observed in the duration of subsequent turns in horses submitted to the *volta sobre patas* manoeuvre can be explained by the complex interaction between neuromuscular activity, biomechanical optimisation, and motor learning. Future studies could address the limitations of this study by employing more advanced biomechanical analysis techniques. Incorporating detailed investigations into muscle activity and biomechanical load distribution would enhance our understanding of the underlying mechanisms of the observed movements. Specifically, examining muscle activation patterns through electromyography (EMG) could provide insights into the timing and intensity of muscle contractions during the manoeuvre. Additionally, a more thorough analysis of joint kinematics using methods such as 3D motion capture could reveal more nuanced details about joint movement and alignment. Investigating metabolic variables could also offer a deeper understanding of the energy demands and efficiency of these movements. As already mentioned, in the present study, the second set of the *volta sobre patas* always lasts longer than the first. Although speed was not measured due to the two-dimensional limitations of the technique, it can be inferred that there is a decrease in this variable between each turn. However, within this context, the data confirm the active participation of the thoracic limbs in pushing the trunk laterally, with greater degrees of abduction and adduction when compared to the pelvic limbs, which in turn, serve as a pivot for the circular movement. The support and suspension times of the appendicular segments demonstrate these active biomechanics during the manoeuvre.

Therefore, in this study, for the first time in the literature on equine biomechanics, the abduction and adduction angles of the appendicular segments were quantitatively characterised, with variations between the thoracic and pelvic limbs and the internal and external sides of the turn. In the forelimbs, abduction angles reached 12.3 ± 4.3° (inner side of the circumference) and 15.4 ± 4.7° (outer side), while adduction angles were 16.7 ± 4.9° (inner side of the circumference) and 12.9 ± 4.4° (outer side). In the hindlimbs, the abduction angles measured were 9.4 ± 3.1° (pivot limb) and 9.9 ± 4.4° (outer side of the circle), while the adduction angles were 11.4 ± 8.2° and 8.7 ± 3.0°, respectively. These objective quantitative records can be used in future studies when focused on the coordination of equine limbs in the sagittal plane during closed circular movements, highlighting the importance of adequate physical preparation in competing animals. These attributes can be amplified by stretching and warming up to improve muscle and joint flexibility, crucial factors for superior athletic performance. Abduction and adduction capacity is closely linked to limb flexibility, directly influencing the effectiveness of circular movements during competitions, such as the *Freio de Ouro*, and can maximise the range of movement and motor coordination, providing animals with a significant competitive advantage.

### 4.3. Limitations of the 2D Kinematic Model for Manoeuvre Analysis

Nicodemus et al. [34] discuss the limitations of a two-dimensional analysis when studying equine tri-dimensional movements. They highlight the challenges of reducing three-dimensional motion to two-dimensional projections, particularly when movements occur outside the sagittal plane. The authors emphasise the importance of considering angular measurements, especially when segmental movement deviates from the plane of projection, potentially distorting angular measurements. This distortion can lead to errors in measurements of adduction/abduction when using a multi-planar analysis (MPA). Consequently, they recommend the use of joint coordinate systems (JCS) or anatomically based coordinate systems when analysing movements beyond the sagittal plane. Additionally, the authors note that the static nature of a global coordinate system (GCS) can affect measurements of adduction/abduction during an MPA analysis, as it fails to dynamically adjust to segment orientations. However, they found that measurements of flexion/extension using an MPA were comparable to those using JCS despite evidence of axial rotation when corrections were made for the displacement plane. Thus, while an MPA may be suitable for assessing flexion/extension, movements beyond the sagittal plane warrant the use of more sophisticated tracking markers and alternative coordinate systems. In the present study, we applied these insights to the analysis of equine movements. The *esbarrada* manoeuvre, characterised by predominantly linear movements and flexion/extension angles, aligned well with the parameters examined by Nicodemus et al. [34], supporting the adequacy of a two-dimensional analysis. However, when analysing the *volta sobre patas*, a more complex and multidirectional movement, we encountered challenges consistent with literature observations. While temporal measurements remained unaffected by the bidimensional nature of the analysis, we recognised the limitations of this approach for capturing the full range of angular motion inherent in the *volta sobre patas*. To address this, angular measurements were selectively taken when the limb was perpendicular to the camera, aiming to minimise potential inaccuracies while acknowledging the need for more advanced analytical methods in future studies of this movement.

Burns and Clayton [35] aimed to provide a detailed description and comparison of the temporal and kinematic characteristics of canter pirouette strides in elite dressage horses, focusing on the differences between pirouette and collected canter movements. The research employed a descriptive approach to analyse the intricate patterns of movement, highlighting the distinct features of pirouette strides compared to collected canter strides. In a similar manner, our study investigated the temporal variables and angles of adduction and abduction of the thoracic and pelvic limbs of Criollo horses performing the *volta sobre patas* manoeuvre, akin to a spin. Despite the methodological variances between our study, which utilised a single 2D camera setup, and the literature, it is noteworthy that an accurate measurement of temporal variables can still be achieved even with a more straightforward data collection approach.

Despite the effectiveness of 2D kinematics as a viable method for the in situ evaluation of movement patterns in horses [36], the presence of factors such as sand elevation during the execution of the *esbarrada* can override the markers, compromising the accuracy of digitisation for the thoracic and, mainly, pelvic joints during movement. This made it impossible to measure the kinematic values of the angular variables of the metatarsophalangeal joint in the hindlimbs. Faced with these challenges, the integration of advanced technologies, such as accelerometers and 3D cinematography, emerges as a promising perspective to overcome the limitations of this technique [37]. These tools could provide a more comprehensive and detailed analysis of the key moments of the *esbarrada* and the sliding stop in horses, offering a more accurate measurement of the quantitative variables related to these manoeuvres in future investigations.

Likewise, the 2D analysis of the *volta sobre patas*, which is essentially a lateralised movement, presents specific challenges that can be improved through the incorporation of 3D video capture technologies. The lateral nature of this movement demands a more comprehensive understanding of the three-dimensional dynamics involved. While two-dimensional videography made it possible to measure extremely important temporal variables as well as adduction and abduction angles at specific moments that the video made possible, 3D cinematography and other technological tools can provide a more complete and detailed view, allowing for more precise measurements of the movement patterns for the axial and appendicular segments during the *volta sobre patas* and homologous movements. Thus, the application of three-dimensional technologies enriches biomechanical analyses, contributing to a broader and more refined understanding of this complex behaviour not only of Criollo breed horses but also of other equestrian disciplines that frequently perform this kind of movement [38,39].

Notwithstanding the inherent limitations of employing 2D kinematic techniques for the analysis of three-dimensional movements, recent studies have shown promising validation of this approach in human research. Demers and Levin [40] investigated the kinematic similarities between reach-to-grasp movements performed in a low-cost 2D virtual environment and those in a physical setting, particularly in healthy individuals and stroke patients, encompassing unilateral and bilateral movements and evaluating various spatial and temporal characteristics. Similarly, Maykut et al. [41] explored the concurrent validity and reliability of 2D video analyses in assessing frontal plane kinematic variables during treadmill running, highlighting challenges such as accurately replicating 3D joint rotations and recognising the potential clinical utility of 2D gait analysis software in studying running kinematics. These studies collectively underscore the evolving validation and applicability of 2D kinematic techniques in human movement research, with potential implications for biomechanical research in equines.

## 5. Conclusions

In the present study, the 2D kinematic analysis of the *esbarrada* and *volta sobre patas* manoeuvres in Criollo breed horses competing in the *Freio de Ouro* provided detailed insights into these complex movements. The determination of values through videography was based on movement analysis models created from their real movement patterns, reflecting the performance at the competition level of this equestrian discipline.

The study found that static goniometry has no association with the protraction angles of the thoracic and pelvic limbs during the moment of engagement in the *esbarrada*. In the *volta sobre patas*, the second set of turns lasts significantly longer than the first, with a greater suspension of both thoracic limbs at both moments. These findings highlight specific kinematic characteristics of Criollo horses in high-level competition, providing valuable data for trainers and breeders aiming to optimise performance.

## Figures and Tables

**Table 1:** Limb joint angles (mean ± SD) (°) of the right (RFL) and left thoracic limbs (LFL) of Criollo breed horses competing in the Freio de Ouro during maximum limb protraction in the esbarrada manoeuvre.

Joint	Limb		RFL and LFL Pooled
	RFL	LFL	
Shoulder (°)	116.0 ± 10.8	119.2 ± 13.4	117.7 ± 6.4
Elbow (°)	129.9 ± 9.9	130.4 ± 14.7	130.2 ± 6.5
Carpal (°)	179.7 ± 4.87	182.0 ± 5.3	180.9 ± 3.4
Forelimb fetlock (°)	220.1 ± 11.0	220.4 ± 10.9	220.3 ± 6.4

RFL: Right forelimb; LFL: left forelimb. N = 31.

**Table 2:** Limb joint angles (mean ± SD) (°) of the right (RHL) and left hindlimbs (LHL) of Criollo breed horses competing in the Freio de Ouro during engagement in the esbarrada manoeuvre.

Joint	Limb		RHL and LHL Pooled
	RHL	LHL	
Lumbosacral (°)	121.3 ± 9.5	126.1 ± 9.4	123.7 ± 5.7
Hip (°)	86.4 ± 9.0	86.9 ± 8.2	86.6 ± 5.0
Stifle (°)	143.5 ± 9.3	143.0 ± 9.0	143.2 ± 9.2
Hock (°)	131.9 ± 13.8	131.7 ± 13.5	131.8 ± 10.0

RHL: Right hindlimb; LHL: left hindlimb. N = 31.

**Table 3:** Temporal kinematic (mean ± SD) (s) variables during volta sobre patas of Criollo breed horses in training for Freio de Ouro.

Variable	*Volta Sobre Patas* Phases					
	Set 1 (M1)	Set 2 (M1)	Total M1	Set 1 (M2)	Set 2 (M2)	Total M2
Total duration (s)	4.4 ± 0.6 ^A^	4.7 ± 0.8 ^B^	9.2 ± 1.4	4.52 ± 0.9 ^a^	4.7 ± 1.0 ^b^	9.2 ± 1.9
Thoracic suspension (s)	0.4 ± 0.2 ^A^	0.4 ± 0.2 ^B^	0.8 ± 0.3	0.41 ± 0.2 ^a^	0.4 ± 0.2 ^b^	0.83 ± 0.3

Set = one 360° spin. Set 1 (M1): Set 1 of the *volta sobre patas* in Moment 1 (s). Set 2 (M1): Set 2 of the *volta sobre patas* in Moment 1 (s). Set 1 (M2): Set 1 of the *volta sobre patas* in Moment 2 (s). Set 2 (M2): Set 2 of the *volta sobre patas* in Moment 2 (s). Thoracic suspension: the total time (s) of both thoracic limbs in the suspension phase. Different letters (A, B and a, b) on the same line within each moment indicate significant differences (*p* ≤ 0.05). N = 31.

**Table 4:** Temporal kinematic values (mean ± SD) (s) for the stance and swing phases of each limb of Criollo breed horses in training for the Freio de Ouro during the volta sobre patas manoeuvre.

Phase	Limb			
	IFL	OFL	PHL	OHL
Stance (s)	2.6 ± 0.3	2.6 ± 0.3	2.9 ± 0.6	2.7 ± 0.4
Swing (s)	1.9 ± 0.6	2.0 ± 0.6	1.7 ± 0.7	1.8 ± 0.6

IFL: inner forelimb; OFL: outer forelimb; PHL: pivot hindlimb; OHL: outer hindlimb. Stance: total support time of the limb during a 360° *volta sobre patas*. Swing = the total suspension time of the limb during a 360° *volta sobre patas*. N = 31.

**Table 5:** The maximum *abduction* and *adduction* angles (°) of each limb (mean ± SD) of Criollo breed horses in training for the Freio de Ouro during the volta sobre patas manoeuvre.

Maximum Angles	Limb			
	IFL	OFL	PHL	OHL
Abduction (°)	12.3 ± 4.6	15.4 ± 4.7	9.4 ± 3.1	9.9 ± 4.4
Adduction (°)	16.7 ± 4.9	12.9 ± 4.4	11.4 ± 8.2	8.7 ± 3.0

IFL: inner forelimb; OFL: outer forelimb; PHL: pivot hindlimb; OHL: outer hindlimb. N = 31.

## Data Availability

The data are not yet on a data repository platform. The data that support the findings of this study are available from the corresponding author, RB, upon reasonable request.

## Supplementary Materials

The following supporting information can be downloaded at: https://www.mdpi.com/article/10.3390/ani14162410/s1. Table S1. Demographic data for individual horses included in the study. Table S2. Kinematics measurements expressed as mean ± SD and standard error (S.E.) of Criollo breed horses competing in the Freio de Ouro during maximum limb protraction in the esbarrada manoeuvre. Table S3. Limb joint angles (mean ± SD and standard error (SE)) (°) of the right (RFL) and left thoracic limbs (LFL) of Criollo breed horses competing in the Freio de Ouro during maximum limb protraction in the esbarrada manoeuvre. Table S4. Limb joint angles (mean ± SD and standard error (SE)) (°) of the right (RHL) and left hind limbs (LHL) of Criollo breed horses competing in the Freio de Ouro during engagement in the esbarrada manoeuvre. Table S5. Temporal kinematic (mean ± SD and standard error (SE)) (s) variables during volta sobre patas of Criollo breed horses in training for Freio de Ouro. Table S6. Temporal kinematic values (mean ± SD and standard error (SE)) (s) for stance and swing phases of each limb of Criollo breed horses in training for Freio de Ouro during the volta sobre patas manoeuvre. Table S7. Maximum abduction and adduction angles (°) of each limb (mean ± SD and standard error (SE)) of Criollo breed horses in training for Freio de Ouro during the volta sobre patas manoeuvre.

## References

[1] Gilbert M., Gillett J. (2012). Equine athletes and interspecies sport. Int. Rev. Sociol. Sport.

[2] Adelman M., Thompson K. (2017). Equestrian Cultures in Global and Local Contexts.

[3] Cardoso C.W. (2022). Além da Cultura: O Negócio do Cavalo Crioulo no Rio Grande do Sul como Emprego e Renda. Undergraduate Thesis.

[4] Galvão E. (1963). O cavalo na América indígena: Nota prévia a um estudo de mudança cultural. Rev. Mus. Paul..

[5] Dowdall R.C. (1982). Criando Criollos.

[6] Vilanova R., Prado F.R.D.A. (2007). Aspectos morfológicos e funcionais em equinos da raça Crioula. Rev. Cient. Elet. Med. Vet..

[7] Cucco D.C., Salles E.L., Santos M.R., Ferreira R., Soriano V.S., Zampar A., Kessler J.D. (2016). Freio de Ouro como ferramenta de seleção na raça crioula. Arch. Zootec..

[8] Abreu H.C.D., La Côrte D., Dessessards F., Brass K.E., Pompemayer E., Luz T.R.R.D., Gasperi D.D. (2011). Claudicação em cavalos Crioulos atletas. Ciênc. Rural.

[9] Silveira B.B., Souza E.C., Dos Santos M.D.N., Porciuncula M.L., Azevedo M.D.S., Duarte C.A., De Souza Junior P. (2020). Digit innervation of the thoracic limb of Criollo horses: Anatomical description and consequences to perineural blocks. Anat. Histol. Embryol..

[10] Góss G.C. Mensuração Ultrassonográfica dos Ligamentos Colaterais da Articulação Interfalangeana Distal de Cavalos da Raça Crioula. Monografia (Specialization—Integrated Residency in Veterinary Medicine)—Federal University of Pampa, Bagé, RS, Brazil, 2017. pp. 13–14. http://dspace.unipampa.edu.br:8080/jspui/handle/riu/4927.

[11] Dalto R. (2006). Freio de Ouro: Uma História a Cavalo.

[12] Pimentel A.M.H., Souza J.R.M., Boligon A.A., Moreira H.L.M., Rechsteiner S.M.D.F., Pimentel C.A., Martins C.F. (2018). Association of morphometric measurements with morphological scores of Criollo horses at Freio de Ouro: A path analysis. Rev. Bras. Zootec..

[13] Torres-Pérez Y., Gómez-Pachón E.Y., Miró-Rodríguez F. (2017). Two-Dimensional Kinematics of Horses at Trot Through Videiomatry and Mathematical Modeling. Rev. Fac. Ing..

[14] Seib I.A., Júnior H.R.M., Nogueira É. (2013). Aceitabilidade da raça crioula em competições de laço comprido em Mato Grosso do Sul: Estudo exploratório. Multitemas.

[15] Rohlf C.M., Garcia T.C., Marsh L.J., Acutt E.V., le Jeune S.S., Stover S.M. (2023). Effects of Jumping Phase, Leading Limb, and Arena Surface Type on Forelimb Hoof Movement. Animals.

[16] Northrop A.J., Hobbs S.J., Holt D., Clayton-Smith E., Martin J.H. (2016). Spatial variation of the physical and biomechanical properties within an equestrian arena surface. Procedia Eng..

[17] Roth I.T., Schielke B., Rensing M., Bernau M. (2021). Comparison of american quarter horses competing in western pleasure, hunter under saddle, and reining using linear traits. Animals.

[18] Alvarez C.G., Rhodin M., Bobbert M.F., Meyer H., Weishaupt M.A., Johnston C., van Weeren P.R. (2006). The effect of head and neck position on the thoracolumbar kinematics in the unridden horse. Equine Vet. J..

[19] Rhodin M., Johnston C., Holm K.R., Wennerstrand J., Drevemo S. (2010). The influence of head and neck position on kinematics of the back in riding horses at the walk and trot. Equine Vet. J..

[20] Kienapfel K. (2015). The effect of three different head–neck positions on the average EMG activity of three important neck muscles in the horse. J. Anim. Physiol. Anim. Nutr..

[21] Rhodin M., Alvarez C.G., Byström A., Johnston C., van Weeren P.R., Roepstorff L., Weishaupt M.A. (2009). The effect of different head and neck positions on the caudal back and hindlimb kinematics in the elite dressage horse at trot. Equine Vet. J..

[22] Jeffcott L.B. (1980). Disorders of the thoracolumbar spine of the horse—A survey of 443 cases. Equine Vet. J..

[23] Townsend H.G.G., Leach D.H. (1984). Relationship between intervertebral joint morphology and mobility in the equine thoracolumbar spine. Equine Vet. J..

[24] Denoix J.M.D. (1999). Spinal biomechanics and functional anatomy. Vet. Clin. N. Am. Equine Pract..

[25] Stubbs N.C., Hodges P.W., Jeffcott L.B., Cowin G., Hodgson D.R., McGowan C.M. (2006). Functional anatomy of the caudal thoracolumbar and lumbosacral spine in the horse. Equine Vet. J..

[26] Denoix J.M. (2014). Muscle groups and their actions: The hindlimb. Biomechanics and Physical Training of the Horse.

[27] Hodson E., Clayton H.M., Lanovaz J.L. (2001). The hindlimb in walking horses: 1. Kinematics and ground reaction forces. Equine Vet. J..

[28] McGowan C.M., Hyytiäinen H.K. (2017). Muscular and neuromotor control and learning in the athletic horse. Comp. Exerc. Physiol..

[29] Todd J.J. (2014). Lactate: Valuable for physical performance and maintenance of brain function during exercise. Biosci. Horiz. Int. J. Stud. Res..

[30] Forestier N., Nougier V. (1998). The effects of muscular fatigue on the coordination of a multijoint movement in human. Neurosci. Lett..

[31] Voge K.R., Dingwell J.B. Relative timing of changes in muscle fatigue and movement coordination during a repetitive one-hand lifting task. Proceedings of the 25th Annual International Conference of the IEEE Engineering in Medicine and Biology Society (IEEE Cat. No. 03CH37439).

[32] Johnston C., Gottlieb-Vedi M., Drevemo S., Roepstorff L. (2010). The kinematics of loading and fatigue in the Standardbred trotter. Equine Vet. J..

[33] Clayton H.M. (2016). Horse species symposium: Biomechanics of the exercising horse. J. Anim. Sci..

[34] Nicodemus M.C., Clayton H.M., Lanovaz J.L. (2008). Comparison of a joint coordinate system versus multi-planar analysis for equine carpal and fetlock kinematics. Comp. Exerc. Physiol..

[35] Burns T.E., Clayton H.M. (2010). Comparison of the temporal kinematics of the canter pirouette and collected canter. Equine Vet. J..

[36] Egan S., Brama P., McGrath D. (2019). Research trends in equine movement analysis, future opportunities and potential barriers in the digital age: A scoping review from 1978 to 2018. Equine Vet. J..

[37] Bragança F.S., Rhodin M., Van Weeren P.R. (2018). On the brink of daily clinical application of objective gait analysis: What evidence do we have so far from studies using an induced lameness model?. Vet. J..

[38] Nora F.G.D.S.A. (2021). Equine biomechanical models for three-dimensional kinematics analysis: Literature review. Braz. J. Dev..

[39] Simonato S.P., Bernardina G.R., Ferreira L.C., Silvatti A.P., Barcelos K.M., Da Fonseca B.P. (2021). 3D kinematic of the thoracolumbar spine in Mangalarga Marchador horses performing the marcha batida gait and being led by hand—A preliminary report. PLoS ONE.

[40] Demers M., Levin M.F. (2020). Kinematic validity of reaching in a 2D virtual environment for arm rehabilitation after stroke. IEEE Trans. Neural Syst. Rehabil. Eng..

[41] Maykut J.N., Taylor-Haas J.A., Paterno M.V., DiCesare C.A., Ford K.R. (2015). Concurrent validity and reliability of 2d kinematic analysis of frontal plane motion during running. Int. J. Sports Phys. Ther..

