# Supplementary material for: 2D Kinematic Analysis of the Esbarrada and Volta Sobre Patas Manoeuvres of Criollo Breed Horses Competing in Freio de Ouro
Source: Animals (Basel). 2024 Aug 20;14(16):2410. doi: 10.3390/ani14162410 (PMC11350687; doi:10.3390/ani14162410)
Supplement: Supplementary file 1 [file animals-14-02410-s001.zip › animals-3095838-supplementary.pdf]

S1. Demographic data for individual horses included in the study

Animal	Age (Years)	Weight (kg)	Height (m)
1	13	400	1.42
2	7	461	1.43
3	7	440	1.45
4	6	440	1.46
5	9	425	1.41
6	7	436	1.43
7	3	420	1.43
8	7	400	1.42
9	6	420	1.43
10	11	410	1.42
11	8	430	1.44
12	4	400	1.42
13	11	420	1.41
14	10	436	1.44
15	5	390	1.42
16	6	430	1.39
17	7	455	1.42
18	9	436	1.42
19	4	436	1.40
20	5	440	1.42
21	6	465	1.44

22	7	450	1.42
23	11	440	1.42
24	7	430	1.42
25	12	390	1.41
26	9	413	1.43
27	5	410	1.39
28	12	445	1.41
29	5	400	1.42
30	8	486	1.48
31	6	440	1.40

S2. Kinematics measurements expressed as mean ± SD and standard error (S.E.) of Criollo breed horses competing in the *Freio de Ouro* during maximum limb protraction in the *esbarrada* manoeuvre.

Variable	Mean±SD	SE
**Sliding length (m)**	4.28 ± 0.99	0.178
**Sliding duration (s)**	1.2 ± 0.1	0.018
**Speed (m/s)**	3.77 ± 0.55	0.099
**Head angle (°)**	81.5 ± 6.91	1.24
**Thoracic limbs protraction (°)**	27.1 ± 2.8	0.50
**Pelvic limbs protraction (°)**	33.0 ± 4.2	0.75

S3. Limb joint angles (mean ± SD and standard error (SE)) (°) of the right (RFL) and left thoracic limbs (LFL) of Criollo breed horses competing in the *Freio de Ouro* during maximum limb protraction in the *esbarrada* manoeuvre

**Joint**	**Limb**				**Two-sided pooled (mean±SD)**	**SE**
	**RFL (mean±SD)**	**SE**	**LFL (mean±SD)**	**SE**		
**Shoulder (°)**	116.0 ± 10.8	1.94	119.2 ± 13.4	2.41	117.7 ± 6.4	1.15
**Elbow (°)**	129.9 ± 9.9	1.78	130.4 ± 14.7	2.64	130.2 ± 6.5	1.17
**Carpal (°)**	179.7 ± 4.87	0.87	182.0 ± 5.3	0.95	180.9 ± 3.4	0.61
**Forelimb fetlock (°)**	220.1 ± 11.0	1.98	220.4 ± 10.9	1.96	220.3 ± 6.4	1.15

RFL: Right forelimb; LFL: left forelimb. N = 31.

S4. Limb joint angles (mean ± SD and standard error (SE)) (°) of the right (RHL) and left hind limbs (LHL) of Criollo breed horses competing in the *Freio de Ouro* during engagement in the *esbarrada* manoeuvre.

Joint	Limb				Two-sided average	SE
	RHL	SE	LHL	SE		
**Lumbosacral (°)**	121.3 ± 9.5	1.71	126.1 ± 9.4	1.69	123.7 ± 5.7	1.02
**Hip (°)**	86.4 ± 9.0	1.62	86.9 ± 8.2	1.47	86.6 ± 5.0	0.90
**Stifle (°)**	143.5 ± 9.3	1.67	143.0 ± 9.0	1.62	143.3 ± 9.2	1.65
**Hock (°)**	131.9 ± 13.8	2.48	131.7 ± 13.5	2.42	131.8 ± 10.0	1.80

RHL: Right hindlimb; LHL: left hindlimb. N = 31.

. **S5.** Temporal kinematic (mean±SD and standard error (SE)) (s) variables during *volta sobre patas* of Criollo breed horses in training for *Freio de Ouro*.

**Variable**	***Volta sobre patas***											
	**Set 1 (M1)**	**SE**	**Set 2 (M1)**	**SE**	**Total M1**	**SE**	**Set 1 (M2)**	**SE**	**Set 2 (M2)**	**SE**	**Total M2**	**SE**
**Total duration (s)**	4.4 ± 0.6[A]	0.11	4.7 ± 0.8[B]	0.14	**9.2 ± 1.4**	**0.25**	4.52 ± 0.9[a]	0.16	4.7 ± 1.0[b]	0.18	**9.2 ± 1.9**	**0.34**
**Thoracic suspension (s)**	0.4 ± 0.2[A]	0.04	0.4 ± 0.2[B]	0.04	**0.8 ± 0.3**	**0.05**	0.41 ± 0.2[a]	0.04	0.4 ± 0.2[b]	0.04	**0.83 ± 0.3**	**0.05**

Set= one 360° spin. Set 1 (M1): Set 1 of *volta sobre patas* in Moment 1 (s). Set 2 (M1): Set 2 of *volta sobre patas* in Moment 1 (s). Set 1 (M2): Set 1 of *volta sobre patas* in Moment 2 (s). Set 2 (M2): Set 2 of *volta sobre patas* in Moment 2 (s). Thoracic suspension: total time (s) of both thoracic limbs in the suspension phase. Different letters (A, B & a, b) on the same line within each Moment indicate significant differences ($p \leqslant 0.05$). N = 31.

S6. Temporal kinematic values (mean±SD and standard error (SE)) (s) for stance and swing phases of each limb of Criollo breed horses in training for *Freio de Ouro* during the *volta sobre patas* manoeuvre.

**Phase**	**Limb**							
	**IFL**	**SE**	**OFL**	**SE**	**PHL**	**SE**	**OHL**	**SE**
**Stance (s)**	2.6 ± 0.3	0.05	2.6 ± 0.3	0.05	2.9 ± 0.6	0.11	2.7 ± 0.4	0.07
**Swing (s)**	1.9 ± 0.6	0.11	2.0 ± 0.6	0.11	1.7 ± 0.7	0.13	1.8 ± 0.6	0.11

IFL: inner forelimb; OFL: outer forelimb; PHL: pivot hindlimb; OHL: outer hindlimb. Stance=:total support time of the limb during a 360° *volta sobre patas*. Swing= total suspension time of the limb during a 360° *volta sobre patas*. N= 31.

S7. Maximum abduction and adduction angles (°) of each limb (mean±SD and standard error (SE)) of Criollo breed horses in training for Freio de Ouro during the volta sobre patas manoeuvre.

Phase	Limb							
	IFL	SE	OFL	SE	PHL	SE	OHL	SE
Abduction (°)	12.3 ± 4.6	0.83	15.4 ± 4.7	0.84	9.4 ± 3.1	0.56	9.9 ± 4.4	0.79
Adduction (°)	16.7 ± 4.9	0.88	12.9 ± 4.4	0.79	11.4 ± 8.2	1.47	8.7 ± 3.0	0.54

IFL: inner forelimb; OFL: outer forelimb; PHL: pivot hindlimb; OHL: outer hindlimb. N= 31.
